# Investigations on Surface Roughness and Tool Wear Characteristics in Micro-Turning of Ti-6Al-4V Alloy

**DOI:** 10.3390/ma13132998

**Published:** 2020-07-06

**Authors:** Kubilay Aslantas, Mohd Danish, Ahmet Hasçelik, Mozammel Mia, Munish Gupta, Turnad Ginta, Hassan Ijaz

**Affiliations:** 1 Department of Mechanical Engineering, Faculty of Technology, Afyon Kocatepe University, 03200 Afyon, Turkey; aslantas@aku.edu.tr; 2 Department of Mechanical and Materials Engineering, University of Jeddah, Jeddah 21589, Saudi Arabia; mdanish@uj.edu.sa (M.D.); hassan605@yahoo.com (H.I.); 3 İscehisar Vocational High School, Afyon Kocatepe University, 03200 Afyon, Turkey; ahascelik@aku.edu.tr; 4 Department of Mechanical Engineering, Imperial College London, Exhibition Road, South Kensington, London SW7 2AZ, UK; m.mia19@imperial.ac.uk; 5 Key Laboratory of High Efficiency and Clean Mechanical Manufacture, Ministry of Education, School of Mechanical Engineering, Shandong University, Jinan 250061, China; munishguptanit@gmail.com; 6 Mechanical Engineering Department, Universiti Teknologi PETRONAS, Perak 32610, Malaysia

**Keywords:** micro turning, surface roughness, material removal rate, RSM, Ti6Al4V alloy, tool wear

## Abstract

Micro-turning is a micro-mechanical cutting method used to produce small diameter cylindrical parts. Since the diameter of the part is usually small, it may be a little difficult to improve the surface quality by a second operation, such as grinding. Therefore, it is important to obtain the good surface finish in micro turning process using the ideal cutting parameters. Here, the multi-objective optimization of micro-turning process parameters such as cutting speed, feed rate and depth of cut were performed by response surface method (RSM). Two important machining indices, such as surface roughness and material removal rate, were simultaneously optimized in the micro-turning of a Ti6Al4V alloy. Further, the scanning electron microscope (SEM) analysis was done on the cutting tools. The overall results depict that the feed rate is the prominent factor that significantly affects the responses in micro-turning operation. Moreover, the SEM results confirmed that abrasion and crater wear mechanism were observed during the micro-turning of a Ti6Al4V alloy.

## 1. Introduction

Micro-mechanical machining, a form of manufacturing used to produce parts with micro dimensions, is noted for higher material removal rates [1,2]. The application of such manufacturing is delicate and requires the use of micro-turning and micro-milling processes. Notable industries are biomedical, defense, aerospace and electronics industries [3]. Though there is a large degree of similarity between traditional turning and micro-turning, turning at the micro scale needs to be accurate as well as precise, causing the necessity for ultra-precision machining [4]. The micro-turning method is especially used in the manufacturing of micro-screws used in orthodontic implants. In this type of small-scale case, the enhancement of surface finish by a secondary method is hard to implement, and thereby process parameters should be chosen so that the desired surface quality is achieved—if possible, the surface finish should be compatible with the grinding process. 

**Figure 1:**
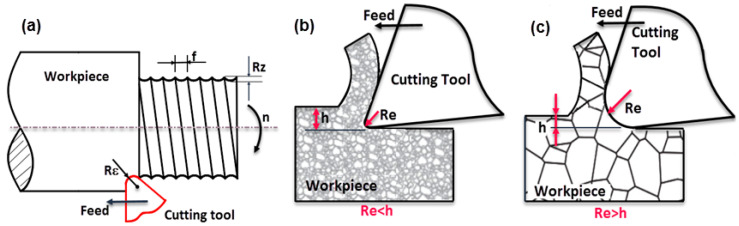
(**a**) Illustration of turning operation showing nose radius (Re), feed rate (*f*), maximum surface roughness (Rz); (**b**) conventional and (**c**) micro-cutting process h: undeformed chip thickness, Re: Edge Radius.

Surface roughness is considered as the prominent parameter that significantly affects the mechanical properties as well as the fatigue strength of the machined part [5]. In addition, the surface roughness values in conventional turning highly depend upon the feed rate. Therefore, the required feed rate for the desired surface quality, the cutting speed and depth of cut are taken into account [6]. On the micro scale, the cutting tool nose radius leaves marks on the workpiece surface (Figure 1a). Depending on the feed value and nose radius, the maximum surface roughness value can be estimated. In conventional turning (Figure 1b), the low feed rate can be indispensable for good surface quality. However, this rule does not always work in micro-turning. In the case of micro-turning (Figure 1c), the feed rate is small enough to be compared to the cutting-edge radius. It has been seen that if the value of feed is smaller than the tool edge radius, an increase in surface roughness can be observed [7,8,9]. The depth of cut has also shown a similar situation. In micro-turning, the depth of cut can approach the workpiece grain size (Figure 1b). This results in both increased thrust force and the deterioration of surface quality.

In conventional turning, a good amount of research effort has been made regarding surface roughness characteristics [10,11,12]. In conventional turning, usually the depth of cut and the feed value are greater than the tool edge and nose radius. Therefore, the difference between the obtained surface roughness value and the theoretical surface roughness value increases. However, in micro-turning, especially in low feed values, the theoretical surface roughness and experimental results do not match. Liu and Melkote [7], while micro-turning the aluminum alloy, established a prediction model for the surface roughness. The effect of plastic side flow was considered in this subjected model. Their model combines cutting parameters and plastic side flow effects with more accurate estimation. Rahman et al. [13] used two different micro-turning techniques for micro-pin production. The responses measured were cutting forces and surface roughness values during the micro-turning operation. The results depict that the average value of surface roughness was 0.1 μm. Alauddin et al. [14] used the second-order polynomials method to establish the surface roughness prediction model. Wang et al. [15] investigated the effect of machining parameters and tool diameter on surface quality in a micro-milling operation. Kuram and Özcelik [16] have developed a model for estimating the surface roughness in the micro-milling process by using a multi-objective optimization technique. The effect of spindle speed, cutting edge radius of the tool, and the roughness of workpiece were investigated. Vipindas et al. [17] examined the surface roughness and top burr formation in the micro-milling of titanium alloy.

In the studies conducted by Aslantas et al. [18] and Ucun et al. [19], the surface roughness and burr width was studied for different process parameters. The result shows that the depth of cut was prominent for surface roughness values, whereas feed per tooth was dominant for burr width. Thepsonthi and Özel [20] optimized the process parameters for surface roughness and burr formation in the micro-milling of a Ti6Al4V alloy. Experiments have been performed and models were obtained by utilizing the particle swarm optimization technique. Kumar [21] also studied the effect of process parameters in the micro-turning process. Cutting speed, depth of cut and feed value were taken as variables, and a C360 copper alloy was used as a workpiece. The analysis shows that the depth of cut is the dominant factor that directly affects the surface roughness and material removal rate values. The influence of cutting parameters on the surface roughness and Material Removal Rate (MRR) is evident from the literature. Optimization of these cutting parameters can greatly help in choosing the optimum cutting parameter for getting the required objective, i.e., minimum surface roughness and high material removal rate. 

Response surface method (RSM) is widely used for developing empirical relations between single and multiple responses [22,23,24]. The most critical factor that affects the output responses can also be determined with this method. Additionally, the multi-response optimization of micro-turning process parameters was performed [25]. Therefore, in this work, the optimization of cutting parameters affecting the surface roughness and MRR was performed in the micro-turning process. Ti6Al4V alloy was used as the workpiece material; average surface roughness of area (Sa) and maximum surface roughness of area (Sz) values were obtained. Single- and multi-objective optimization with the objective of obtaining minimum surface roughness and maximum MRR were carried out by utilizing the response surface methodology. The most dominant factor which affects the surface roughness was also identified. In the end, the SEM was performed on used tools to understand the wear behavior values.

## 2. Materials and Methods

### 2.1. Workpiece and Cutting Tool Material

**Figure 2:**
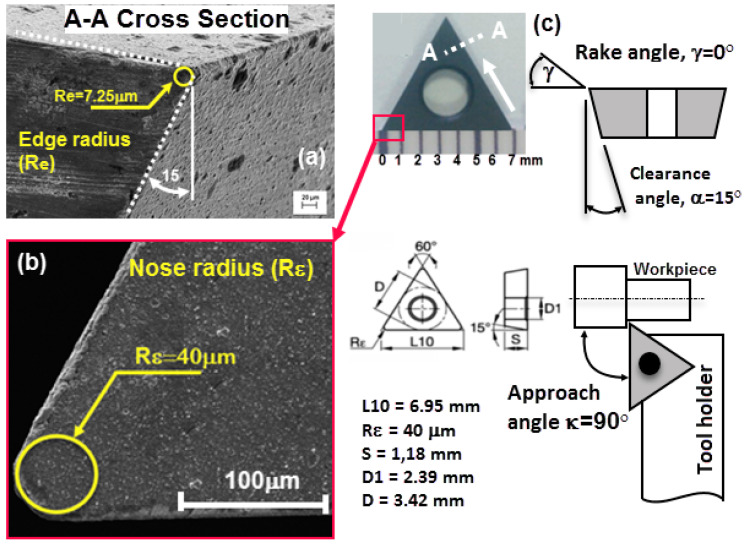
(**a**) Edge radius (**b**) nose radius (**c**) other dimensions of the cutting tool used in micro turning process.

The Ti6Al4V alloy is preferred as the workpiece. This titanium alloy, known as Grade 5, is especially used as an implant material in the biomedical sector. It also has a wide usage area as a screw in dental implant applications. The alloy used in the study was annealed after the manufacturing process and no aging was done. The chemical compositions of the Ti6Al4V alloy are shown in Table 1 and the mechanical properties are given in Table 2. The machining operation was conducted using the cutting tool received from the Kennametal 2-µm-coated tool (ISO name TDHB07T12S0). It has a rake angle of 0°, approach angle of 90° and clearance angle of 15° in the machining condition, nose radius of 40 µm, and edge radius of 7.25 µm (See Figure 2).

### 2.2. Experimental Setup

**Figure 3:**
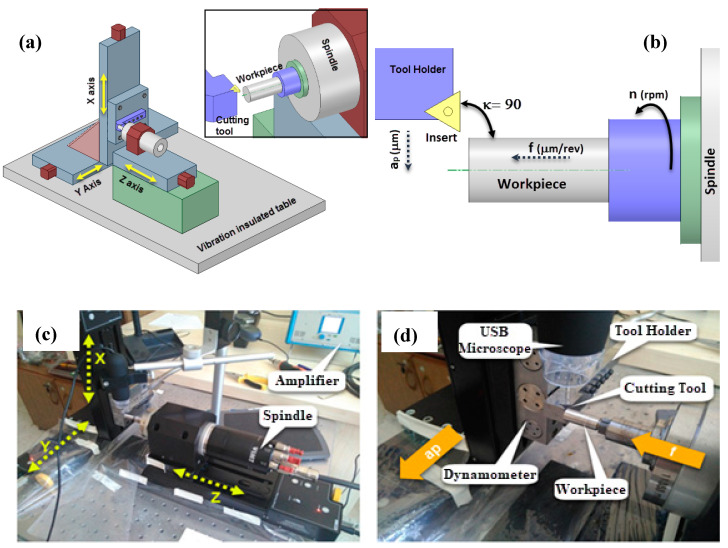
(**a**) Schematic representation of micro-turning setup; (**b**) position of cutting tool and workpiece relative to each other; (**c**) axis definitions of experimental setup; (**d**) close view of cutting setup.

Figure 3 shows the experimental setup—a specialized setup for high-precision cutting with high speed. The highest speed achievable is 60,000 rpm, and the highest travel distance of guideway is 150 mm, maintaining a repeatability of 0.4 μm. The cutting tool was placed on the mini dynamometer that is fixed to the x-axis. The feed was applied along the z-axis and the depth of cut was applied along the y-axis. The approach angle of the cutting tool was 90° and a USB microscope was used to more clearly observe the cutting zone in the experiments. The system was maintained as vibration free by using an optical table. A constant cutting distance (75 mm) was used in experiments to observe the effects of cutting parameters and to eliminate the effects of tool wear.

### 2.3. Surface Roughness Measurement

**Figure 4:**
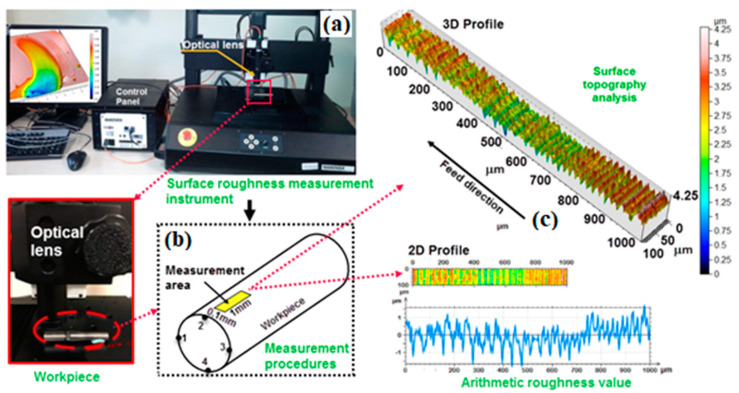
Measurement procedure of surface roughness in current work, (**a**) surface roughness tester; (**b**) area under consideration for the roughness measurement; (**c**) 3D surface topography of the area.

The representative index for the surface quality after the machining was granted as the surface roughness parameters. These parameters were measured using Nanovea optical profilometer, which works with white light technology. In Figure 4a, the surface roughness tester is shown. The table where the sample is placed is movable in the x and y axis directions and the focusing distance of the optical lens is adjusted with the z axis. Scanning was performed on a 1 × 0.1 mm area (Figure 4b). The surface roughness was measured at four different points with 90° angles on each workpiece. In this study, Sa and Sz values were measured as the surface roughness and taken into consideration. A three-dimensional surface topography of a surface is shown in Figure 4c.

In metal-cutting operations, the material removal rate is expressed as the chip volume removed in one minute and MRR can be calculated by using Equation (1) [26]

(1) $$Q=V_{c}\times f\times a_{p}$$

where *Q* is the MRR in mm^3^/min, *V_c_* is the cutting speed, *f* is feed rate and *a_p_* is depth of cut. MRR is an indication of how slow or fast the machining speed works. It is an important performance parameter for micro-machining. In micro-machining (especially micro-milling), a high MRR value results in high surface roughness, rapid tool wear, and burr formation. It is important to determine the maximum MRR value without compromising surface quality and for longer tool life.

### 2.4. Design of Experiment

The analysis consisted of determining the influence of each control factor on the surface roughness parameters found after the micro-turning operation. For that purpose, the control factors were defined first, as can be seen in Table 3. As we can see, the feed rate, the cutting speed and the depth of cut have three levels of values. Afterward, these values are oriented among themselves to create the overall design for the experimentation. This was carried out using a face centered composite design; details can be found in Reference [20]. The process parameters with their different levels are given in Table 3. Respective to each experiment, the measurement was conducted. The collected data were then used for the further analysis, using complete a manual of response surface methodology (RSM)—mathematical modeling, analysis and optimization.

### 2.5. Response Surface Methodology

RSM is a complete package for the mathematical modeling, statistical analysis and optimization of the single or multiple responses within the framework of multiple inputs [27,28]. Here, the input parameters, i.e., speed, feed and depth of cut are analyzed to derive the relationship of inputs with the surface roughness parameters (Sa and Sz). Although linear, as well as second-order, polynomial relations can be formed, based on the literature knowledge, it is found that for machining responses, the second-order polynomial relationship works effectively. A general second-order relation is shown in Equation (2)

(2) $$Y=\beta_{0}+\overset{p}{\underset{i=1}{\sum }}\beta_{i}X_{i}+\overset{p}{\underset{i=1}{\sum }}\beta_{ii}X_{i}^{2}+\overset{p-1}{\underset{i=1}{\sum }}\overset{p}{\underset{j=1}{\sum }}\beta_{ij}X_{i}X_{j}$$

Here, *β*_0_ is the constant. The coefficient *βi* is the coefficient term for the linear terms, *β_ii_* are coefficients for the square term of the variables, and the *β_ij_*, are the coefficients for the interacting terms. 

## 3. Results and Discussions

According to the objectives defined in the introduction section, the experimental results and analysis are given in this section, under a number of sub-sections. Initially, the data found from experiments were collected. Then, the subsequent analysis is reported. For this, full quadratic models were constructed, and then analysis of variance, which shows the influence of each factor on the responses, and, finally, the responses were optimized. Note that significant terms were identified respective to a statistical significance of 0.05. This has been done for the model as well as for the single terms (*V_c_*, *f* and *a_p_*), square terms (*V_c_^2^*, *f^2^* and *a_p_^2^*) and interaction terms (*V_c_f*, *V_c_a_p_* and *fa_p_*). The F-value was marked for the relative influence determination. Table 4 lists the experimental results respective to the 20 experiments, which were oriented as per the description of the design of the experiment. Besides the surface roughness parameters for the micro-turning operation, the material removal rate was considered (calculated). 

### 3.1. Model of Average Roughness (Sa) 

As the first step, the analysis of variance is conducted for the average surface roughness (Sa) and shown in Table 5. It should be noted that, respective to each source, the sum of square term, the degree of freedom term, the mean square, F-value and *p*-value are listed. It can be seen that the model is acceptable, as the F-value is quite high and the *p*-value is less than 0.05. Therefore, the model is statistically significant. Likewise, the feed rate and the depth of cut have been found to be statistically significant. The square term for speed and depth of cut, and the interaction terms for feed-depth of cut were also significant. It can be said that the other terms were statistically insignificant. Based on the F-values, it is possible to claim that the feed rate has the highest value, therefore it is most dominant factor, followed by the influence of depth of cut, then it’s square term. 

The arithmetic model developed for average roughness (Sa) is given by Equation (3).

(3) $$\begin{array}{ll} Sa= & -1.78\times 10^{-3}\;Vc+0.0225f+0.0335ap+3.325\times 10^{-6}Vc^{2}-5.64\times 10^{-5}f^{2}\\ & -1.31\times 10^{-3}ap^{2}-1.39\times 10^{-6}Vcf+1.34\times 10^{-5}Vcap-3.77\times 10^{-4}fap+0.29 \end{array}$$

This model, however, includes significant as well as non-significant terms. This means that more refinement is required for this model to improve the model efficiency. This can be done by the removal of non-significant terms by using backward elimination; however, those terms which are required for hierarchy are exempted. After doing this, the new model for ANOVA for average surface roughness is shown in Table 6. This new model then can be analyzed for comparing the R^2^.

It is appreciable that the F-value of the new model was increased to 84.73 from 56.69. The *p*-value was found to be under 0.05, which is an indication for a significant model. Interestingly, it is shown that the *V_c_^2^*, being a non-significant term, is still in the model. This term is kept to maintain the hierarchy. After such refinement, the *R*^2^ values are compared before and after the backward elimination, listed in Table 7. 

It is to be noted that the overall *R*^2^ and adjusted *R*^2^ remained the same and they are very close to unity; however, the predicted *R*^2^ value increased from 0.85 to 0.92. Moreover, the adequate precision value increased. This indicates that the refinement of the model improved the efficiency. As such, the final model for Sa, which was used for further analysis and optimization, is shown by Equation (4).

(4) $$\begin{array}{ll} Sa= & -1.505\times 10^{-3}Vc+0.0193f+0.0383ap+3.114\times 10^{-6}Vc^{2}-1.354\\ & \times 10^{-3}ap^{2}-3.775\times 10^{-4}fap+0.257 \end{array}$$

### 3.2. Model for Maximum Roughness Height (Sz)

The maximum roughness height (Sz) has also been analyzed statistically to develop the model. For that purpose, the ANOVA was performed and all important values are given in Table 8. 

The model *p*-value was under 0.05. For roughness parameters, based on *p*-value criteria, most of the terms are statistically non-significant, except three terms (*f*, *a_p_* and *a_p_^2^*). As such, it is imperative to refine the model by using backward elimination. That has been done here, and the new model is listed in Table 9. 

It is admissible that the new mode *p*-value is less than 0.05 and less than the previous *p*-value, hence it is significant and obviously improved. In this refined model, the F-value shows that the feed rate is the most dominant followed by the square term of depth of cut. Nevertheless, it is necessary to compare the *R*^2^ parameters in Table 10. Table 10 shows that the overall *R*^2^ value decreased from 0.91 to 0.84. As such, the model has lack of fitness. It can also be noticed that the adjusted *R*^2^ value of the model was closer for the backward elimination compared to the primary model. This shows that the model efficiency was increased. Equation (5), the mathematical model of the maximum height roughness, was achieved and used for further computation and optimization.

(5) $$\begin{array}{ll} Sz= & 6.53\times 10^{-3}Vc+0.2202f+0.40895ap-3.555\times 10^{-6}Vc^{2}-8.444\times 10^{-4}f^{2}\\ & -0.012ap^{2}-1.605\times 10^{-4}Vcf-1.658\times 10^{-4}Vcap\\ & -2.092\times 10^{-3}fap-0.93 \end{array}$$

### 3.3. Adequacy Tests

**Figure 5:**
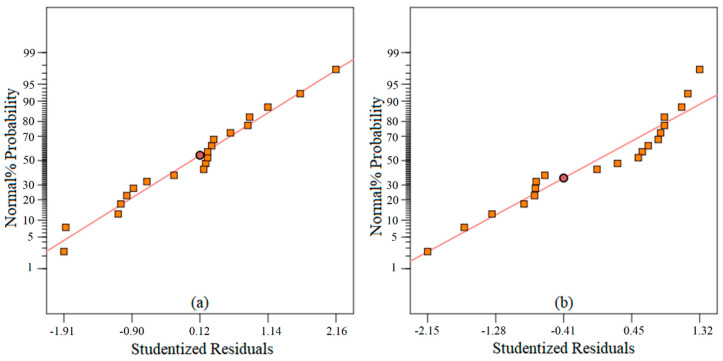
Residual plots for the model developed for (**a**) average surface roughness (Sa); (**b**) maximum roughness height (Sz).

**Figure 6:**
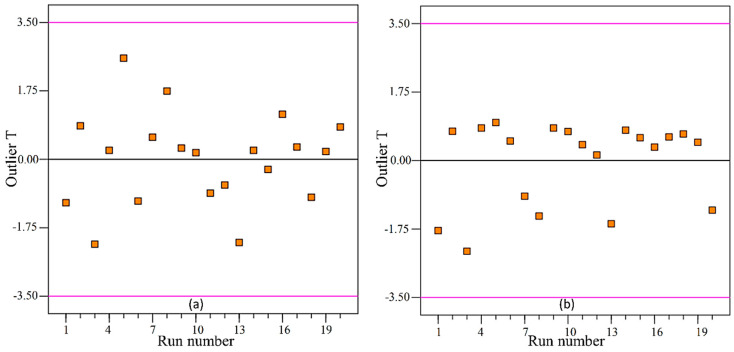
Outlier plot for (**a**) average roughness (Sa); (**b**) maximum roughness height (Sz).

The constructed models were put into trial for the adequacy test. It is noted that the residual plot of the data should not follow any type of trend. The residual plots for both Sa and Sz are shown in Figure 5. As it is visible the datapoints for both plots follow a straight-line path and are free from any trend or sequence—an indication that the model is adequate for further analysis, i.e., prediction model and optimization. However, further investigation is required to be certain about the complete adequacy of the models. Thereafter, the models were tested for abnormality—if the datapoints shifted on either side or were distributed fairly on the both sides with respect to the reference line. For that purpose, the outlier’s plots were constructed and shown in Figure 6. As a rule, if the data fall outside the ±3.5 permissible range, then they are considered outliers, i.e., abnormal data. Interestingly, all the datapoints for the present study were found within the data range of permissibility. Therefore, the models can be claimed as adequate.

### 3.4. Experimental Verification Test

**Figure 7:**
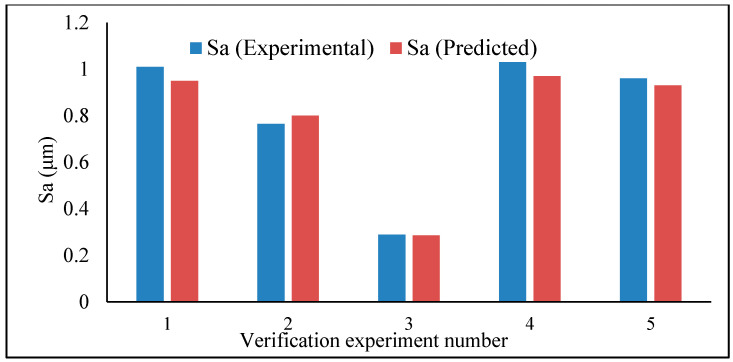
Average roughness parameter (*Sa*) – verification test.

**Figure 8:**
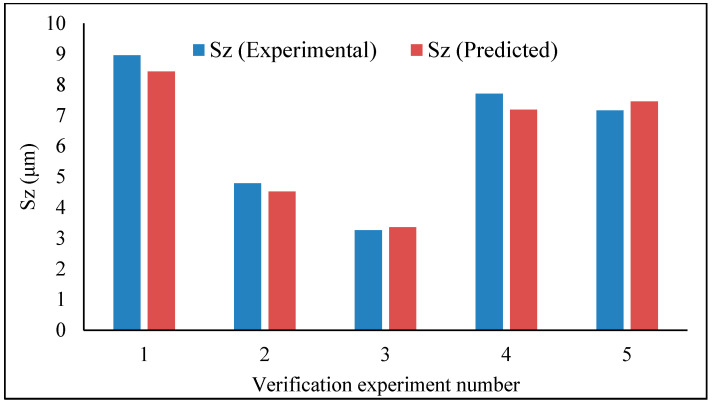
Maximum roughness height (*Sz*) – verification test.

The prediction models were developed and tested for verification. This has been done in five random experimental sets of data. For each set, the experimental as well as the predicted data are plotted side by side, as can be seen in Figure 7 (average roughness parameter) and Figure 8 (maximum height roughness parameter). Interestingly, the agreement between the predicted value and the experimental value is quite reasonable, and therefore the models can be accepted. However, the model of average roughness parameter showed better accuracy in the prediction—from 1% to 5.94% error, while that of the maximum height surface roughness parameter ranges from 3.07% to 6.8%.

### 3.5. 3D Response Surface, One Factor Plots and Analysis by SEM

**Figure 9:**
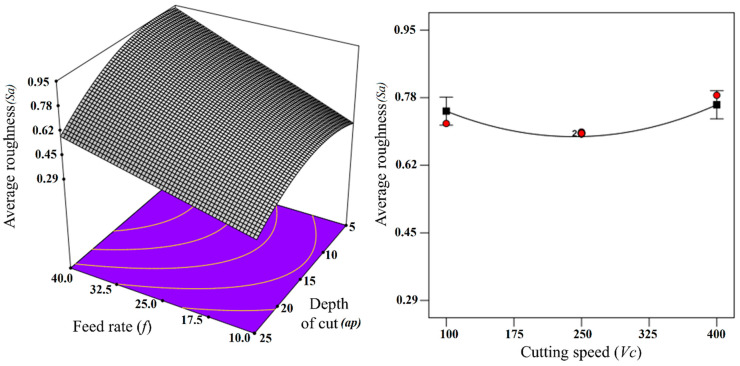
Average surface roughness with 3D plot and one factor plot.

**Figure 10:**
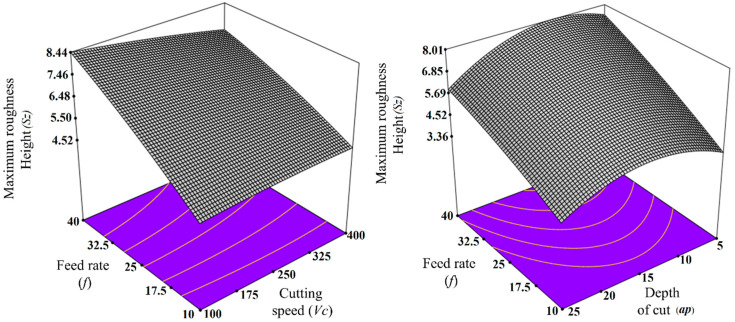
Maximum roughness height with 3D plot and one factor plot.

**Figure 11:**
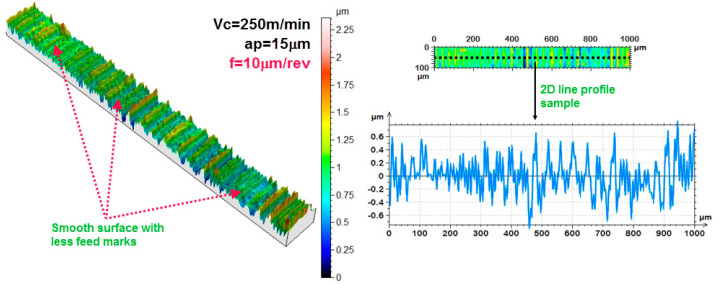

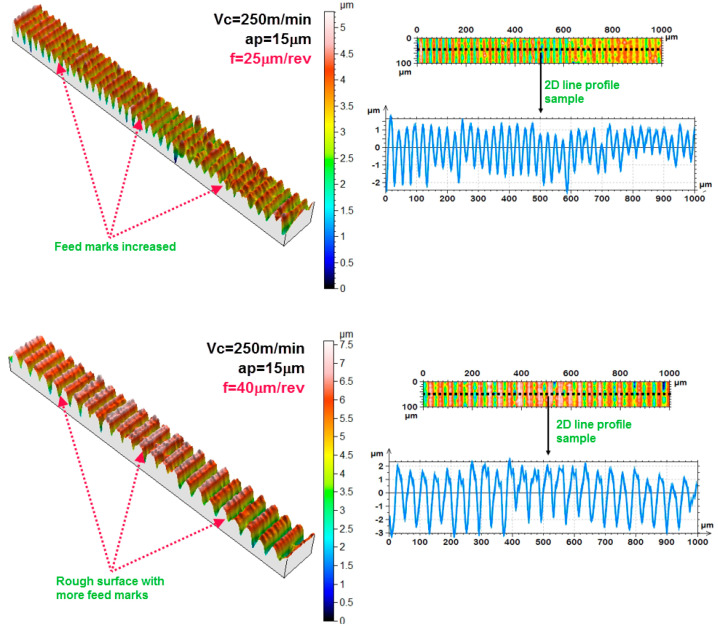
Influence of feed rate on surface 2D and 3D surface profiles.

**Figure 12:**
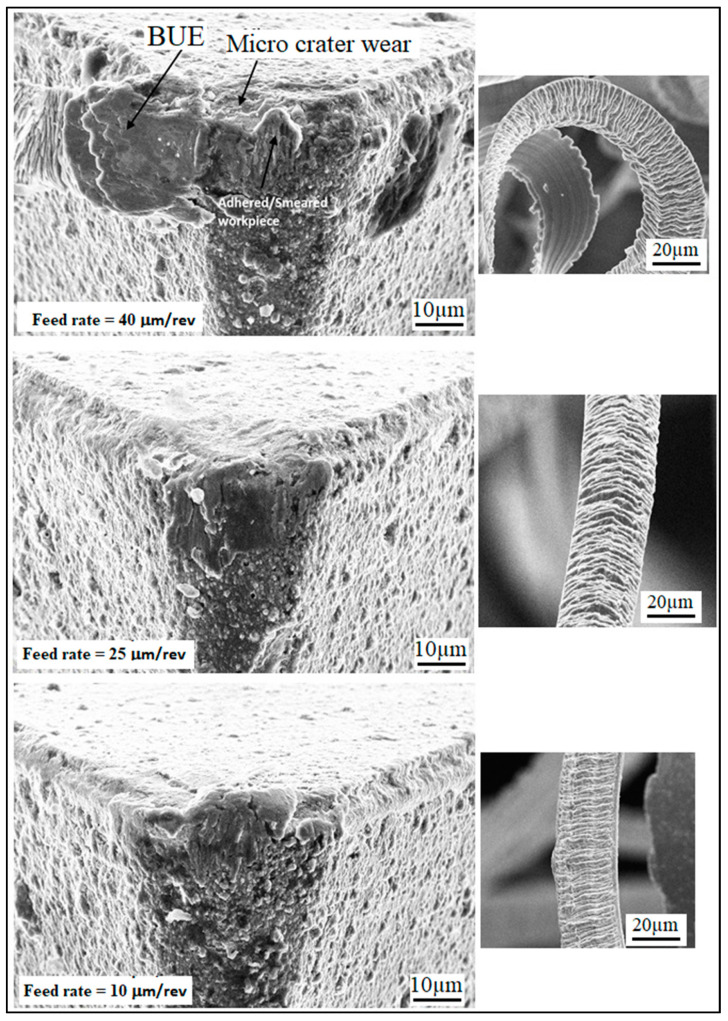
SEM images showing the effect of feed rate on the tool and chip morphology at *V_c_* = 250 m/min and a*_p_* = 15 µm.

At this stage, 3D response surface plots and one-factor plots were plotted (as shown in Figure 9 and Figure 10) and the effects of cutting parameters on the response were analyzed. The information extracted from the plots was also verified with the experimental results, such as the 3D surface profiles of the tested specimens and scanning electron micrographs (SEM) of the tool and chips. The 3D response surface plot showing the effect of input parameter on the response (average roughness parameter and maximum height roughness parameter) has been shown in Figure 9 and Figure 10. As is evident from Figure 9 and Figure 10, both Sa and Sz increases with every increment in the Feed rate. This trend can be verified by the 3D profiles of the machined surface obtained for varying feed rate, which are shown in Figure 11. It is quite clear from Figure 11 that not only Sa but also Sz become higher for higher values of feed rates. For more details, SEM images for the tool, together with the chips, were also obtained for the varying feed rates, as shown in Figure 12. 

**Figure 13:**
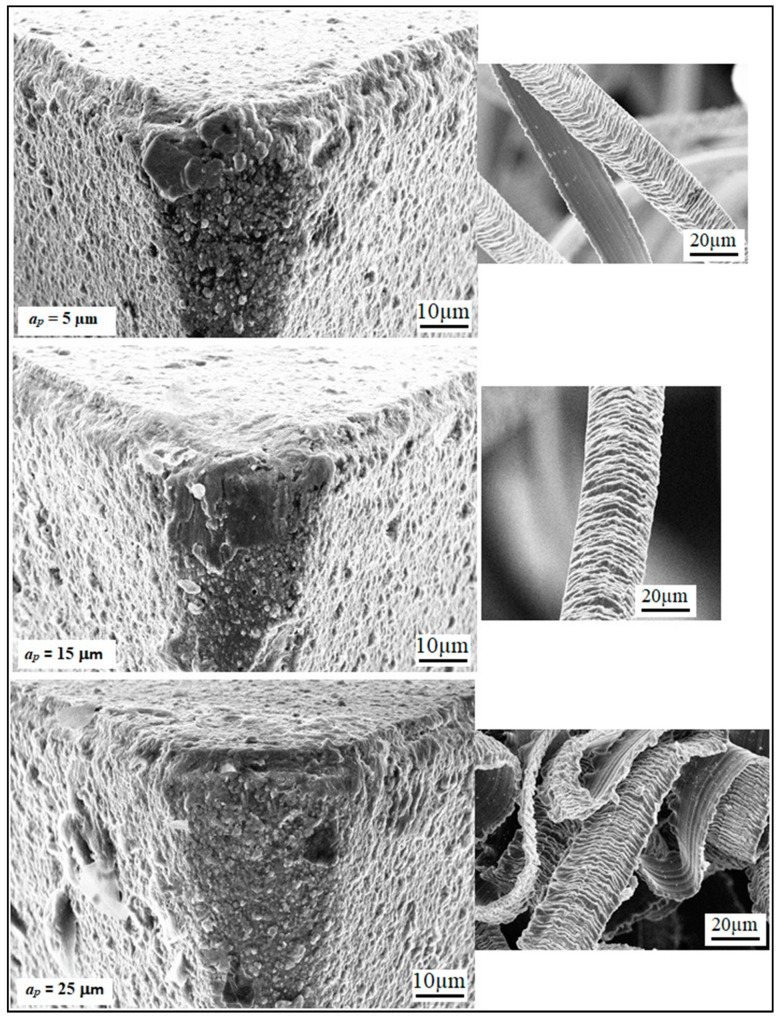
SEM images showing the effect of depth of cut on the tool and chip morphology at *V_c_* = 250 m/min and feed rate = 25 µm/rev.

At a lower feed (*f* = 5 µm/rev), the adhered work material can be seen on the tool tip, but the tool wear is not significant, thereby giving lower values of surface roughness parameters (Sa and Sz). At *f* = 15 µm/rev, the increase in crater wear on the tool can be observed and that could be the reason for the increase in the surface roughness values. However, for *f* = 25 µm/rev, a significant increase in crater wear and also in the adhered work material was observed, as shown in Figure 12. The built-up edges (BUE) on the tool were also observed at this feed rate. All these factors have contributed to the poor surface finish values (higher Sa and Sz values) at this point. The chips’ morphology was more or less the same for every variation in the feed rate, as shown in Figure 12. The serrated chips were observed for the cases, however, the serration was clearer at a lower feed rate value. It can be concluded from the SEM images that the lower feed rate values will be best for micro-machining in the present scenario, which is in full agreement with the empirical model and 3D response plots obtained by the RSM method. The effect of cutting speed (*V_c_*) on the Sa factors was found to be mixed, as shown in Figure 9. It was observed that the value of Sa tends to decrease with increases in the cutting speed, but it rises again with further increases in *V_c_*. The effect of cutting speed on the Sz value is not significant, as shown in Figure 10. The depth of cut (*a_p_*) has a significant effect on the surface roughness values (Sa and Sz), which is evident from the surface roughness plots shown in Figure 9 and Figure 10. For both cases, it was observed that surface roughness values first tend to increase when the depth of cut is increased from 5 to 15 µm. However, further rises in the depth of cut tend to produce lower values of surface roughness parameters. This trend was also supported by the SEM images taken for tool and chips for different depths of cut (5 to 15 µm), which are shown in Figure 13. The crater wear on the tool was not significant, except for the depth of cut = 15 µm, which can be observed in Figure 13. As can be seen from the SEM photographs of Figure 12 and Figure 13, tool wear is minimal and BUE and chip plastering occurs mainly at the tool tip. The nose radius is almost unchanged. Therefore, the change in surface roughness is affected by BUE and chip plaster, not by tool wear. 

The adhered work material was highest when the depth of cut was 15 µm. These factors may be the reason for the higher surface roughness observed at this point. A significant amount of work material adhered on the tool was also noticed when the depth of cut was only 5 µm. This may be due to the ploughing effect that can occur at a very low depth of cut, which is not desirable for a machining operation as it increases the possibility of tool wear, thereby producing unacceptable surface finish [29]. Therefore, it can be concluded here that, for good surface quality results, the depth of cut should be practiced in the higher range, as this is supportive of both the productivity as well as the finish surface roughness.

### 3.6. Optimization of Surface Roughness Parameters and Material Removal Rate

Two surface roughness parameters and the material removal rate have been considered for the system optimization—a multi-objective optimization. For that purpose, the composite desirability approach has been granted. Its details can be found in Reference [30,31,32]. The common desirability function is presented in Equation (6).

(6) D=(d1×d2×d3……..×dn)1n=(∏i=1ndi)1n

Here, the desirability has been represented by the *d_i_*, and the responses are represented by n. Depending on the condition, each response should either have a low value or high value. As the highest is the better value, the desirability is defined as Equation (7).

(7) $$\{\begin{array}{c} d_{i}=0\;if\;response<low\;value\\ 0\leq d_{i}\leq 1\;if\;response\;in\;between\;low\;and\;high\;value\\ d_{i}=1\;if\;response>high\;value \end{array}$$

However, if the target is to minimize the response, the desirability function becomes Equation (8).

(8) $$\{\begin{array}{c} d_{i}=1\;if\;response<low\;value\\ 0\geq d_{i}\geq 1\;if\;response\;in\;between\;low\;and\;high\;value\\ d_{i}=0\;if\;response>high\;value \end{array}$$

Four optimization cases (two single-objective and two multi-objective) were considered in the present analysis.

- For minimum Sa;
- For minimum Sz;
- For minimization of both Sa and Sz simultaneously;
- For minimum of surface roughness (Sa and Sz) and maximum MRR at the same time.

The input variables, and the responses listed with the goal of optimization, their lower limits and upper limits and the respective importance, are shown in Table 11. 

The target for the multi-objective optimization is to finalize a solution that is supportive of the best possible outcomes from all three responses. The main objective for the multi-objective optimization here is to gain the optimum solution for which minimum surface roughness and maximum MRR can be obtained simultaneously. The solutions for the multi-objective optimization, i.e., minimum surface roughness (Sa and Sz) and maximum MRR case, are summarized in Table 12.

**Figure 14:**
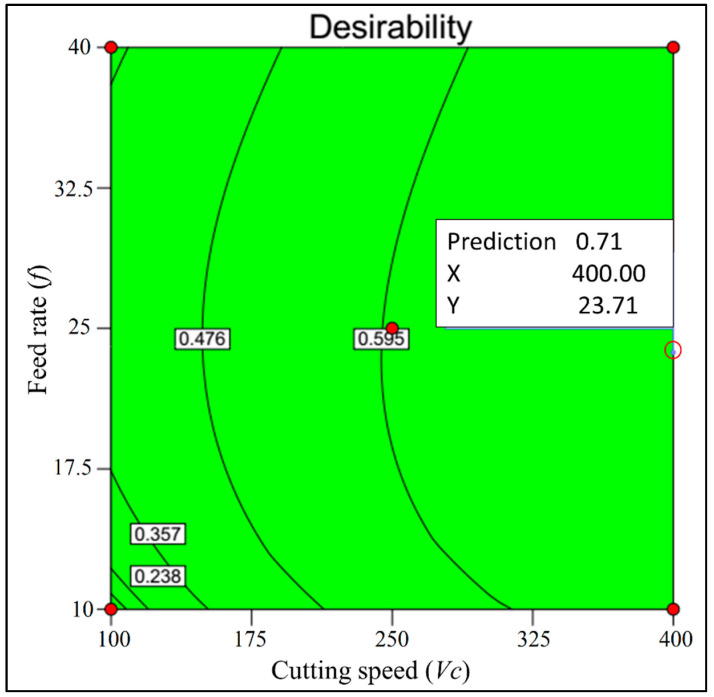
Contour graph for the multi-objective optimization.

**Figure 15:**
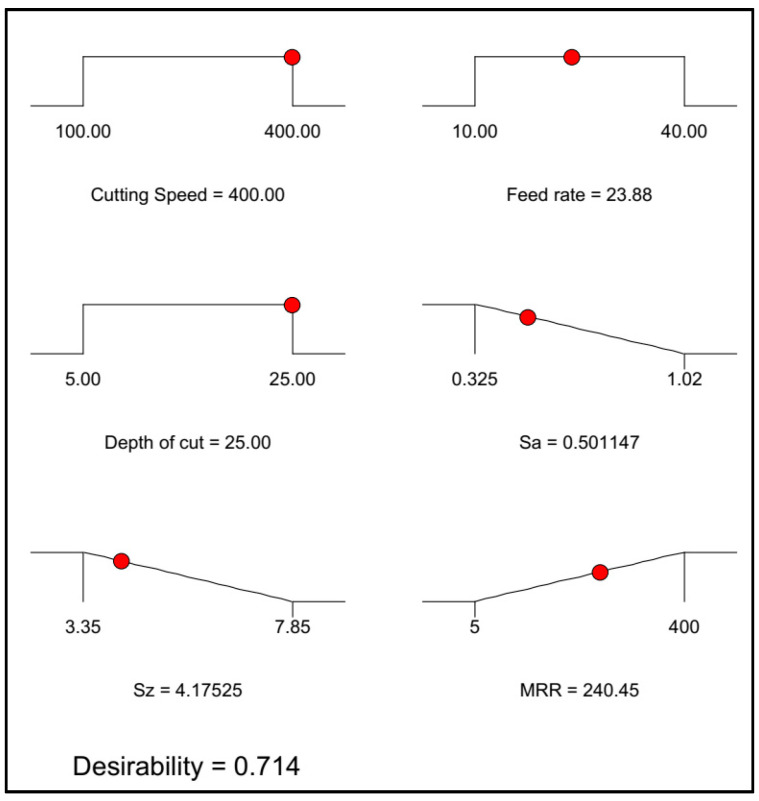
Multi-objective optimization solution as ramp function.

From Table 12, it is suggested that the best possible solution obtained here has a desirability of 0.714. The respective solution is a cutting speed of 400 m/min, feed rate of 23.71 μm/rev and depth of cut of 25 microns. The optimum average surface roughness parameter is 0.50 μm, the optimum maximum height roughness parameter is 4.16 μm and optimum material removal rate is 239.03 mm^3^/min. The contour plot and ramp function plot respective to the optimum solution is shown in Figure 14 and Figure 15, respectively. Moreover, the solutions for all the cases are listed in Table 13. 

The combinations varied depending on the selectivity of the response. For instance, when the two roughness parameters were considered, leaving the material removal rate out of consideration, the desirability was 1.0 when the cutting speed was 340.49 m/min, feed rate was 10.24 μm/rev and the depth of cut was 24.87 μm.

## 4. Conclusions

1. Empirical relations between cutting parameters and surface roughness (*Sa* and *Sz*) of the TiAl4V alloy was successfully developed using RSM for the micro-turning process;
2. The efficiency of both models was checked according to the different *R*^2^ terms. The developed models showed good accuracy in terms of correlation coefficient, close to unity. The residual plots and the outliers plot showed the adequacy of the models. Last but not least, the verification test showed superior accuracy, an error value of less than 7% for both the average roughness parameter and maximum height roughness parameter;
3. With the increase in feed rate, both the *Sa* and *Sz* of the TiAl4V alloy were found to be increased, while a mixed trend was observed for other cutting parameters. Overall, the most dominant factor which affects the *Sa* and *Sz* of the micro-turned TiAl4V was found to be the feed rate;
4. The tool wear results show that the crater wear is the dominant wear for micro-turned Ti-6Al-4V alloys. Moreover, the higher serrations in the chips were observed at high feed rate values, which is also the reason for the poor surface roughness values;
5. All optimization results are as follows:
  - Minimum *Sa* optimization: *V_c_* = 156.14 m/min, *f* =10.44 μm /rev and *a_p_* = 24.92 μm;
  - Minimum *Sz* optimization: *V_c_* = 339.67 m/min, *f* =10.55 μm /rev and *a_p_* = 24.87 μm;
  - Minimum *Sa* and *Sz* optimization: *V_c_* = 340.49 m/min, *f* = 10.24 μm /rev and *a_p_* = 24.87 μm;
  - For minimum of surface roughness (*Sa* and *Sz*) and maximum *MRR* at the same time: *V_c_* = 400 m/min, *f* = 23.71 μm/rev and *a_p_* = 25 μm;
6. The optimized values for *Sa*, *Sz* and *MRR* obtained by the multi-objective optimization approach were 0.50 μm, 4.16 μm and 239.03 mm^3^/min, respectively.

## Figures and Tables

**Table 1:** Ti6Al4V alloy chemical composition (% by weight).

Element	Al	V	Fe	C	O	N	H	Ti
Wt %	6.40	4.16	0.16	0.028	0.154	0.017	0.001	Balance

**Table 2:** Mechanical properties of Ti6Al4V Alloy.

Properties	Value
Tensile Strength (MPa)	900–1000
Yield Strength (MPa)	830–910
Elongation (%)	10–18
Elastic Modulus (GPa)	114
Hardness (Brinell)	330–340

**Table 3:** Process variables used in cutting tests.

Levels	Cutting Speed (*V*_*c*_) (m/min)	Feed Rate (*f*) (μm/rev)	Depth of Cut (*a*_*p*_) (μm)
1	100	25	5
2	250	10	15
3	400	40	25

**Table 4:** Experimental results on Sa, Sz and material removal rate (MRR) for different cutting parameters.

Sr. NO	Inputs			Outputs		
	Cutting Speed (*V_c_*) (m/min)	Feed Rate (*f*) (μm/rev)	Depth of Cut (a_p_) (μm)	Average Roughness (Sa) (μm)	Maximum Roughness Height (Sz) (μm)	Material Removal Rate (mm^3^/min)
1	100.00	25.00	15.00	0.72	5.94	37.50
2	400.00	10.00	25.00	0.39	3.35	100.00
3	250.00	10.00	15.00	0.42	3.83	37.50
4	250.00	25.00	15.00	0.70	6.98	93.75
5	100.00	10.00	5.00	0.52	3.48	05.00
6	100.00	40.00	25.00	0.62	6.87	100.00
7	250.00	40.00	15.00	0.91	7.48	150.00
8	250.00	25.00	25.00	0.48	4.23	156.25
9	250.00	25.00	15.00	0.70	6.98	93.75
10	250.00	25.00	15.00	0.69	6.93	93.75
11	400.00	40.00	5.00	0.99	7.12	80.00
12	400.00	40.00	25.00	0.64	5.02	400.00
13	250.00	25.00	5.00	0.62	5.06	31.25
14	250.00	25.00	15.00	0.70	6.95	93.75
15	400.00	10.00	5.00	0.48	4.07	20.00
16	100.00	40.00	5.00	1.02	7.85	20.00
17	250.00	25.00	15.00	0.70	6.85	93.75
18	100.00	10.00	25.00	0.33	3.63	25.00
19	250.00	25.00	15.00	0.69	6.77	93.75
20	400.00	25.00	15.00	0.79	5.59	150.00

**Table 5:** Table of ANOVA for average surface roughness (Sa).

Source	Sum of Squares	Degree of Freedom	Mean Square	*F*-Value	Dominance of Factor	*p*-Value
Model	0.657	9	0.072	56.69	99.55%	<0.0001
*V_c_*	6.084 × 10^−4^	1	6.084 × 10^−4^	0.48	0.09%	0.5040
*f*	0.42	1	0.42	330.95	63.64%	<0.0001
*a_p_*	0.14	1	0.14	108.67	21.21%	<0.0001
*V_c_^2^*	0.015	1	0.015	12.16	2.27%	0.0059
*f^2^*	4.423 × 10^−4^	1	4.423 × 10^−4^	0.35	0.07%	0.5676
*a_p_^2^*	0.047	1	0.047	37.09	7.12%	0.0001
*V_c_* *f*	7.812 × 10^−5^	1	7.812 × 10^−5^	0.062	0.01%	0.8088
*V_c_* *a_p_*	3.240 × 10^−3^	1	3.240 × 10^−3^	2.56	0.49%	0.1407
*f a_p_*	0.026	1	0.026	20.26	3.94%	0.0011
Residual	0.013	10	1.266 × 10^−3^	-	1.97%	-
Total	0.66	19	-	-	100%	-

**Table 6:** Table of ANOVA for average roughness (Sa) after refinement.

Source	Sum of Squares	Degree of Freedom	Mean Square	F-Value	Dominance of Factor	p-Value
Model	0.64	6	0.11	84.73	96.97%	<0.0001
*V_c_*	6.084 × 10^−4^	1	6.084 × 10^−4^	0.48	0.09%	0.4999
*f*	0.42	1	0.42	331.71	63.64%	<0.0001
*a_p_*	0.14	1	0.14	108.92	21.21%	<0.0001
*V_c_^2^*	0.016	1	0.016	12.44	2.42%	0.0037
*a^2^*	0.059	1	0.059	46.47	8.94%	<0.0001
*f a_p_*	0.026	1	0.026	20.31	3.94%	0.0006
Residual	0.016	13	1.263 × 10^−3^	-	2.42%	-
Total	0.66	19	-	-	100%	-

**Table 7:** *R*^2^ parameter for average roughness (Sa) model before and after the backward elimination.

Parameter	Before	After
*R*^2^ (overall)	0.98	0.98
Adjusted *R*^2^	0.96	0.96
Predicted *R*^2^	0.85	0.92
Adeq Precision	27.44	31.37

**Table 8:** ANOVA table for the maximum roughness height (Sz).

Source	Sum of Squares	Degree of Freedom	Mean Square	*F*-Value	Dominance of Factor	*p*-Value
Model	40.00	9	4.44	11.29	91.03%	0.0004
*V_c_*	0.69	1	0.69	1.74	1.57%	0.2161
*F*	25.54	1	25.54	64.87	58.12%	<0.0001
*a_p_*	2.01	1	2.01	5.10	4.57%	0.0475
*V_c_^2^*	0.018	1	0.018	0.045	0.04%	0.8368
*f^2^*	0.099	1	0.099	0.25	0.23%	0.6264
*a_p_^2^*	3.96	1	3.96	10.06	9.01%	0.0100
*V_c_* *f*	1.04	1	1.04	2.65	2.37%	0.1345
*V_c_* *a_p_*	0.50	1	0.50	1.26	1.14%	0.2883
*f a_p_*	0.79	1	0.79	2.00	1.80%	0.1876
Residual	3.94	10	0.39	-	8.97%	-
Total	43.94	19	-	-	100%	-

**Table 9:** ANOVA table for the maximum roughness height (Sz) after the backward elimination process.

Source	Sum of Squares	Degree of Freedom	Mean Square	F-Value	Dominance of Factor	*p*-Value
Model	36.82	3	12.27	27.57	83.80%	<0.0001
*f*	25.54	1	25.54	57.37	58.13%	<0.0001
*a_p_*	2.01	1	2.01	4.51	4.57%	0.0497
*a^2^*	9.28	1	9.28	20.84	21.12%	0.0003
Residual	7.12	16	0.45	-	16.20%	-
Total	43.94	19	-	-	100%	-

**Table 10:** *R*^2^ parameter for maximum roughness height (Sz) model before and after the backward elimination.

Parameter	Before	After
*R* ^2^	0.91	0.84
Adjusted *R*^2^	0.83	0.81
Predicted *R*^2^	0.61	0.73
Adequate Precision	10.73	16.78

**Table 11:** Inputs, outputs, ranges and the importance for the optimization.

Name	Goal	Lower Limit	Upper Limit	Importance
Cutting speed (*Vc*)	is in range	100	400	-
Feed rate (*f*)	is in range	10	40	-
Depth of cut (*ap*)	is in range	5	25	-
Average roughness *(Sa)*	Minimize	0.325	1.02	5
Maximum roughness height *(Sz)*	Minimize	3.35	7.85	5
Material removal rate *(MMR)*	Maximize	5	400	5

**Table 12:** Multi-objective optimization solution.

Sr. No.	*Vc*	*f*	*a_p_*	*Sa*	*Sz*	*MMR*	Desirability
1	400.00	23.71	25.00	0.50	4.16	239.03	0.714 Selected
2	400.00	23.88	25.00	0.50	4.17	240.45	0.714
3	400.00	23.18	25.00	0.49	4.13	234.525	0.713
4	400.00	22.24	24.97	0.49	4.08	226.261	0.712
5	400.00	22.56	24.94	0.49	4.11	228.645	0.710
6	400.00	33.41	25.00	0.59	4.70	321.503	0.700
7	400.00	10.31	25.00	0.37	3.16	125.099	0.659
8	100.00	10.00	5.01	0.47	3.21	27.4834	0.356

**Table 13:** List of solutions for the combinations of target objectives.

Optimization Cases	*V_c_* (m/min)	*f* (μm/rev)	*a_p_* (μm)	*Sa* (μm)	*Sz* (μm)	*MMR* (mm^3^/min)	Desirability
Minimum *Sa*	156.14	10.44	24.92	0.32	-	-	1.000
Minimum *Sz*	339.67	10.55	24.87	-	3.34	-	1.000
Minimization of both *Sa* and *Sz*	340.49	10.24	24.87	0.32	3.30	-	1.000
Minimization of *Sa* and *Sz* and maximization of *MMR*	400.00	23.71	25.00	0.50	4.16	239.03	0.714

## References

[1] Wu D., Wang B., Fang F. (2019). Effects of tool wear on surface micro-topography in ultra-precision turning. Int. J. Adv. Manuf. Technol..

[2] Wojciechowski S., Matuszak M., Powałka B., Madajewski M., Maruda R.W., Królczyk G.M. (2019). Prediction of cutting forces during micro end milling considering chip thickness accumulation. Int. J. Mach. Tools Manuf..

[3] Yousuff C.M., Danish M., Ho E.T.W., Basha K., Hussain I., Hamid N.H.B. (2017). Study on the optimum cutting parameters of an aluminum mold for effective bonding strength of a PDMS microfluidic device. Micromachines.

[4] Boswell B., Islam M.N., Davies I.J. (2018). A review of micro-mechanical cutting. Int. J. Adv. Manuf. Technol..

[5] Danish M., Ginta T.L., Abdul Rani A.M., Carou D., Davim J.P., Rubaiee S. (2019). Investigation of surface integrity induced on AZ31C magnesium alloy turned under cryogenic and dry conditions. Procedia Manuf..

[6] Piotrowska I., Brandt C., Karimi H.R., Maass P. (2009). Mathematical model of micro turning process. Int. J. Adv. Manuf. Technol..

[7] Liu K., Melkote S.N. (2006). Effect of plastic side flow on surface roughness in micro-turning process. Int. J. Mach. Tools Manuf..

[8] Zhang T., Liu Z., Shi Z., Xu C. (2013). Size effect on surface roughness in micro turning. Int. J. Precis. Eng. Manuf..

[9] Zhao M., He N., Li L., Liu Z.Q., Wu W.F., Zheng K.B. (2012). Analyses of Size Effect on Surface Roughness in Micro Turning Process. In Materials Science Forum. Trans Tech. Publ..

[10] Danish M., Yasir M., Mia M., Nazir K., Ahmed T., Rani A.M.A. (2020). High speed machining of magnesium and its alloys. High Speed Mach.

[11] Mia M., Morshed M.S., Kharshiduzzaman M., Razi M.H., Mostafa M.R., Rahman S.M.S., Ahmad I., Hafiz M.T., Kamal A.M. (2018). Prediction and optimization of surface roughness in minimum quantity coolant lubrication applied turning of high hardness steel. Measurement.

[12] Mia M., Dhar N.R. (2016). Prediction of surface roughness in hard turning under high pressure coolant using Artificial Neural Network. Measurement.

[13] Rahman M.A., Rahman M., Kumar A.S., Lim H.S., Asad A. (2006). Development of micropin fabrication process using tool based micromachining. Int. J. Adv. Manuf. Technol..

[14] Alauddin M., El Baradie M.A., Hashmi M.S.J. (1996). Optimization of surface finish in end milling Inconel 718. J. Mater. Process. Technol..

[15] Wang X., Lu X., Jia Z., Jia X., Li G., Wu W. (2013). Research on the prediction model of micro-milling surface roughness. Int. J. Nanomanuf..

[16] Kuram E., Ozcelik B. (2013). Multi-objective optimization using Taguchi based grey relational analysis for micro-milling of Al 7075 material with ball nose end mill. Measurement.

[17] Vipindas K., Kuriachen B., Mathew J. (2019). Investigations into the effect of process parameters on surface roughness and burr formation during micro end milling of TI-6AL-4V. Int. J. Adv. Manuf. Technol..

[18] Aslantas K., Ekici E., Çiçek A. (2018). Optimization of process parameters for micro milling of Ti-6Al-4V alloy using Taguchi-based gray relational analysis. Measurement.

[19] Ucun İ., Aslantaş K., Gökçe B., Bedir F. (2014). Effect of tool coating materials on surface roughness in micromachining of Inconel 718 super alloy. Proc. Inst. Mech. Eng. Part B J. Eng. Manuf..

[20] Thepsonthi T., Özel T. (2012). Multi-objective process optimization for micro-end milling of Ti-6Al-4V titanium alloy. Int. J. Adv. Manuf. Technol..

[21] Kumar S.P.L. (2019). Measurement and uncertainty analysis of surface roughness and material removal rate in micro turning operation and process parameters optimization. Measurement.

[22] Danish M., Yahya S., Saha B.B. (2019). Modelling and optimization of thermophysical properties of aqueous titania nanofluid using response surface methodology. J. Therm. Anal. Calorim..

[23] Keshtegar B., Mert C., Kisi O. (2018). Comparison of four heuristic regression techniques in solar radiation modeling: Kriging method vs RSM, MARS and M5 model tree. Renew. Sustain. Energy Rev..

[24] Danish M., Ginta T.L., Habib K., Carou D., Rani A.M.A., Saha B.B. (2017). Thermal analysis during turning of AZ31 magnesium alloy under dry and cryogenic conditions. Int. J. Adv. Manuf. Technol..

[25] Chabbi A., Yallese M.A., Meddour I., Nouioua M., Mabrouki T., Girardin F. (2017). Predictive modeling and multi-response optimization of technological parameters in turning of Polyoxymethylene polymer (POM C) using RSM and desirability function. Measurement.

[26] Groover M.P. (2007). Fundamentals of Modern Manufacturing: Materials Processes, and Systems.

[27] Singh G., Gupta M.K., Mia M., Sharma V.S. (2018). Modeling and optimization of tool wear in MQL-assisted milling of Inconel 718 superalloy using evolutionary techniques. Int. J. Adv. Manuf. Technol..

[28] Mia M., Singh G., Gupta M.K., Sharma V.S. (2018). Influence of Ranque-Hilsch vortex tube and nitrogen gas assisted MQL in precision turning of Al 6061-T6. Precis. Eng..

[29] Popov A., Dugin A. (2015). Effect of uncut chip thickness on the ploughing force in orthogonal cutting. Int. J. Adv. Manuf. Technol..

[30] Mia M. (2017). Multi-response optimization of end milling parameters under through-tool cryogenic cooling condition. Measurement.

[31] Gupta M.K., Sood P.K., Sharma V.S. (2016). Optimization of machining parameters and cutting fluids during nano-fluid based minimum quantity lubrication turning of titanium alloy by using evolutionary techniques. J. Clean. Prod..

[32] Gupta M.K., Mia M., Singh G.R., Pimenov D.Y., Sarikaya M., Sharma V.S. (2019). Hybrid cooling-lubrication strategies to improve surface topography and tool wear in sustainable turning of Al 7075-T6 alloy. Int. J. Adv. Manuf. Technol..

